# ‘This diarrhoea is not a disease …’ local illness concepts and their effects on mothers’ health seeking behaviour: a qualitative study, Shuhair, Yemen

**DOI:** 10.1186/1471-2458-14-581

**Published:** 2014-06-10

**Authors:** Hana H Webair, Abdulla S Bin Ghouth

**Affiliations:** 1 Department of Family Medicine, Hadhramout University, Almukalla, Hadhramout, Yemen; 2 Department of Community Medicine, Hadhramout University, Almukalla, Hadhramout, Yemen

**Keywords:** Children, Illness concept, Health seeking behaviour

## Abstract

**Background:**

Globally, about seven million children under the age of five died in 2011. Local illness concepts are thought to be related to inappropriate health-seeking behaviour, and therefore, lead to child mortality. The aim of this study was to contribute to the definition of common local illness concepts with their effects on health-seeking behaviour for common childhood illnesses.

**Methods:**

A qualitative focus group study was conducted between April 1 and 6, 2013. Participants were drawn purposefully from the vaccination unit at Shuhair Health Centre in Yemen. Four focus group discussions were conducted. The total number of participants was 31 mothers with at least one child under the age of five with a history of fever, diarrhoea, cough, or difficulty breathing during the 14 days preceding the study. Data was collected and analysed using micro-interlocutor analysis.

**Results:**

The mean age of the participants was 31 years (SD ± 4). There was remarkable concordance in local illness concepts across the focus groups. During focus group discussions, six local illness concepts (Senoon, lafkha, halib, didan, raqaba, and ayn) were mentioned. Local illness concepts determined the type of treatment. Most of these illnesses were not treated medically. Lafkha, halib, raqaba, and ayn were always classified as “not for medical treatment”, whereas senoon and didan as sometimes “not for medical treatment”. For medical symptoms, i.e. fever, diarrhoea, cough, and difficulty breathing, medical therapy was usually an option; these were classified as never or sometimes “not for medical treatment”. Mothers trust in traditional medicine and believe that it is always beneficial and never harmful. The participants do not disclose traditional medicine use with their doctors because doctors oppose these practices and are not open enough to these types of treatment.

**Conclusions:**

Local illness concepts for common child illnesses are widespread, and they determine the type of treatment used. Interventions to improve children’s health should use local illness concepts to educate parents. Traditional medicine as a treatment option in primary care should be considered.

## Background

Worldwide, about seven million children under the age of five died in 2011. More than half of these deaths were due to preventable conditions, mainly pneumonia, preterm birth complications, diarrhoea, birth asphyxia and malaria [1]. However, health services alone are not sufficient to reduce child mortality; success requires cooperation between health workers and families, supported by the community [2].

Improving parents’ health seeking behaviour (HSB) could contribute in reducing infant mortality in developing countries [3] provided that adequate quality care is available at healthcare facilities [4]. HSB has been defined as any action taken by a person who has a health problem or illness in order to find a proper remedy [5].

A recent study in Yemen (Shuhair) about the factors affecting HSB [6] by Webair & Bin-Gouth found that considering the illness as not for medical treatment was one of the main reasons for avoiding or delaying seeking medical care for ill children.

However, this study did not explore these concepts. So, the local illness concepts should be considered when developing national disease control programs [7].

Although there are a countless number of studies that focused on complementary and alternative medicine [8-11], only few published studies were found about local illness concepts and traditional medicine [7,12-17]. The majority of those studies were qualitative or combined quantitative and qualitative. The main difference between traditional and complementary medicine is that the former refers to a set of health care practices that are part of that country’s own tradition [18]. In Yemen, Bamatraf [12] described some of the traditional illness beliefs related to diarrhoea in children and concluded that mothers had more confidence in folkloric treatment than in the medical services and these practices led to avoidance or delaying seeking medical care for many children. A study in Ghana [16] identified the local illness classification system and found it to be a barrier against seeking medical care.

Gilman et al. [14] described traditional illness concepts among Laotian Refugees and found that they used to integrate traditional healing beliefs and practices with the use of modern health services. Many studies found that patients prefer to use a combination of both medical and traditional healing measures in response to their illnesses [12-14]. Some African hospitals integrate the use of both traditional medicine as well as standard medical care. By this approach, they fully took into account the cultural realities of the populations for which they are responsible [19].

Yemen is in need of improving its socioeconomic and health situation. Under-five mortality rate in Yemen is as high as 77/1000 live births and it is one of the highest in the region [20]. The Millennium Development Goals (MDGs) have been the most successful global anti-poverty measures in history. Millennium Development Goal 4 (MDG4) target is to reduce by two thirds, between 1990 and 2015, the under-five mortality rate [21]. Yemen has insufficient progress towards MDG4 [22]. As diarrhoea and pneumonia were the main killers for children under the age of five, the current study focused on the symptoms which are the common presentations of diarrhoea and pneumonia.

This study explores the local illness concepts, with identification of their effects on the mothers’ HSB for childhood fever, diarrhoea, cough, and difficulty of breathing (DOB). At the same time, it aims to determine mothers’ attitude toward both traditional and modern medicine, to ultimately understand how to design effective policies to improve HSB and child health.

## Methods

### Design and setting

As the current study aimed to explore and explain people’s actual thoughts and beliefs related to childhood illnesses, focus group discussions were a good method to achieve this objective because they encourage participants to talk freely and discuss topics related to their children. It also would help to develop appropriate messages for educational interventions. The study research questions are: What are the common local concepts related to childhood illnesses in Shuhair City? How do local illness concepts affect health seeking behaviour for common childhood illnesses in Shuhair City? The focus groups were conducted in Shuhair, which is a small, ancient city in Hadhramout Governorate in Yemen. Shuhair was selected because the local illness concepts were expected to be widespread and to negatively affect HSB, despite the availability of health services at a health centre [6].

The respondents were recruited from the vaccination unit of Shuhair Health Centre, which is the main source of health care in the city and one of the training centres for the master program of family medicine sponsored by Hadhramout University, College of Medicine. Visitors to the SHC vaccination unit were selected because they all have children under the age of five and are not seeking medical care for illnesses, but rather, vaccination. Another important point is that the vaccination coverage in SHC is more than 90%, which indicates that women come from almost all areas of Shuhair, making the sample more representative of the overall population [6]. Mothers who are not inhabitants of Shuhair City were excluded because they may not be aware about the local concepts.

### Participants

The participants were recruited purposefully between April 1 and 6, 2013, from 9:00 AM to 10:30 AM. A female community health worker approached mothers who had a child below five years of age with a history of fever, diarrhoea, cough, and/or DOB during the preceding 14 days when they entered the vaccination unit. She explained the focus group discussion and obtained verbal informed consent and contact information until the required number of participants was reached. Four focus group discussions were conducted, with 31 mothers participating; three groups contained eight women, and the fourth contained seven. The participants ranged in age from 20 to 38 years, with a mean age of 31 (SD ± 4); all of the women were married. One woman was illiterate, 19 had primary school education, and 11 had secondary school education.

### Data collection and analysis

The focus groups met in a Shuhair Health Centre discussion room. A female family physician acted as a moderator who facilitated, encouraged, and controlled discussions, and a female community health worker acted as an observer and took notes during the discussions. Audio- and videotaping were not used because of cultural issues and refusal of the respondents. Both the moderator and the observer had training in focus group discussions.

The discussions explored three questions for each symptom separately: fever, diarrhoea, cough, and DOB. The questions were: “What are the causes of the illness?”, “What do you do when your child has this illness?”, and “What is your opinion about traditional and modern medicine?” Other additional questions were asked according to the direction of the discussion. Probing, rephrasing, reminder questions and hypothetical questions were used to encourage and control the discussions. A full focus group debrief was conducted after each session. The observer, the first author, and the second author were present for this meeting.

The discussions were documented and analysed using micro-interlocutor analysis, because it enables the disclosure of information regarding the level of consensus/disagreement. It helps the researcher to treat each focus group member as a unique and important study participant and provides both quantitative and qualitative information with a great deal of nonverbal language [23].

In this method, the observer took notes using paper and pencil, writing the responses in a matrix. The matrix included the questions in the rows and the participants in one column each. The responses included agreement or disagreement, manifested by both verbal and nonverbal language. Non-response was also documented. Quotes which gave answers to focus group questions and have agreed upon by several participants or that summarised statements repeated across groups were selected.

Both authors discussed the details of the method of data analysis. The first author wrote all of the results, which were then checked by the second author. Several versions were produced based on the comments from both authors. An independent doctor who has experience in focus group discussion analysis revised the results and helped in resolving disagreements.

The results were calculated with Statistical Product and Service Solutions (SPSS) version 20. For each question, the answer was entered as a variable, and the response of the mother was documented as “1” if she agreed, “2” if she disagreed, or “0” if there was no response. Demographic characteristics of the respondents were described using frequencies, mean, and standard deviation (SD).

### Definitions

Traditional medicine is the sum total of the knowledge, skills, and practices based on the beliefs and experiences indigenous to different cultures (whether explicable or not) used in the maintenance of health as well as in the prevention, diagnosis, or treatment of illnesses [18]. “Modern healers” means qualified medical professionals.

Traditional healers are non-qualified persons who treat sick children using traditional medicine methods such as massage, herbs, and cauterization. Drug sellers are nonqualified persons who sell drugs without medical prescriptions.

### Ethical considerations

The Department of Family Medicine of Hadhramout University reviewed and approved the study protocol. A simple and clear explanation of the research aims and procedure were provided to the SHC manager and persons involved in the study.

Informed consent was obtained from the manager and staff members included in the study, and feedback was returned to them. Similarly, verbal consent was obtained from all of the mothers who participated in the discussion. The privacy and confidentiality of the respondents were ensured. For evidence of adherence to RATS guideline for qualitative manuscripts see Additional file 1.

## Results

After completing four focus group discussions, the researchers agreed that a saturation level of ideas had been reached. The demographic data of the participants are detailed in Table 1.

**Table 1:** Demographic characteristics of participants

**Variable**	**No.**	**%**
Age		
20-	5	16.12
25-	12	38.70
30-40	14	45.16
Educational level		
Illiterate	1	3.23
Primary school	19	61.29
Secondary school	11	35.48
No. of offspring		
≤ 2		10	32.26
> 2	21	67.74	
Address			
Level one^a^	20	64.52	
Level two or three^b^	11	35.48	

^a^Level one includes areas near the health center according to the division of the catchment areas for immunization activities [6].

^b^Level two and three include areas away from the health center according to the division of the catchment areas for immunization activities [6].

### Fever

The participants defined fever as “hotness of body”. The mothers attributed fever to bacteria, hot weather, inflammation, sore throat, or senoon, which is a traditional illness concept believed to be caused by teething (Table 2).

**Table 2:** Local illness concepts, classification, and treatment

**Concept**	**Definition**	**Classification**	**Treatment**
*Senoon*	It is believed to be caused by teething manifested by fever, with or without diarrhea, and chest problems, associated with poor appetite, hotness of child’s head, abrading and redness of gums, and excessive salivation	Sometimes not for medical treatment	Nothing or drugs
*Lafkha*	It is thought to be caused by having a Demon inside of child, or when the child comes under the power of a demon if he/she sits alone for a long time or goes outside during the night. It results in abnormal and strange behavior, loud crying and shouting, and hitting heads on the wall or ground.	Always not for medical treatment	Qur’an reading by Ashaikh^a^ as it expels the Demons out.
*Halib*	It is believed to be caused by dietary errors, manifested by watery diarrhea with or without vomiting	Always not for medical treatment	*Ghutrah*^*b*^
*Didan*	It is thought to be caused by worms and parasites. It is manifested by loss of appetite, nausea, vomiting, abdominal pain, with changes in the composition of stool and foul smell.	Sometimes not for medical treatment	Herbs or drugs
*Raqaba*	It is believed to be caused by falling down, manifested by vomiting and extreme tiredness with loss of appetite with or without fever and frequent stool.	Always not for medical treatment	*Tarqeeb*^*c*^
*Ayn*	It is an Islamic concept believed to be caused by an evil person’s gaze (eye) that is loaded with admiration which harms whoever is looked at, manifested by a prolonged illness of any type that fails to respond to any medical or traditional treatment	Always not for medical treatment	Qur’an and pouring the wash-water of the eye-giver on the eye receiver

^a^*Ashaikh* is a religious healer.

^b^*Gutrah* is hot oil applied to soles, umbilicus and possibly, the head to treat halib.

^c^*Tarqeeb* is massage done in a specific manner by a traditional healer or the grandmothers for *raqaba.*

The mothers differentiated senoon fever from other types of fever. When asked how one could determine that the cause of fever was senoon, one participant (a 25-year-old mother of two children under the age of five with a secondary education) replied,

*“Fever of senoon makes the child’s palms and soles very hot, and is high grade and continuous. It responds poorly to syrup and wet sponges and does not respond to treatment given by doctors; they do not realize what senoon is. Suppositories are effective”*.

A 31-year-old mother with a secondary school education and first-aid training looked surprised and said that senoon fever could be relieved by syrup, and that if it is not relieved, she takes her child to the health centre. Thirty of the 31 mothers agreed upon not seeking medical care for senoon fever except if it becomes very severe, because it is not a disease. A 35-year-old mother intervened: *“Senoon causes fever. For some children it also causes diarrhoea, while for others it attacks the chest”*. All seven mothers in the group agreed by smiling, head nodding, or saying *“yes”.*

All of the respondents used to treat fever by drugs and/or a wet sponge, but some (15 out of 31) avoid washing the child because they thought it would lead to fits. Twenty seven mothers used hair cream with water and lemon on the child’s body. Fourteen mothers stated that if the child is shivering or feels cold they cover the child completely with a blanket. The 31 mothers came to an agreement regarding taking their children to modern healers if the fever persists for more than two days, the child develops fits, or the child becomes unconscious.

### Diarrhoea

The mothers defined diarrhoea as frequent or watery stool. Child diarrhoea was attributed to bacteria, dirt, eating a lot of sweets, halib, didan, raqaba, and senoon.

Didan, raqaba, and halib are traditional illness concepts (Table 2). Halib was believed to be caused by diet errors, didan was thought to be caused by worms and parasites, and raqaba is a traditional illness concept believed to be caused by falling down. The mothers (27 of 31) agreed that senoon-related diarrhoea is recognized by the stool being very offensive, the child has excessive salivation, the child abrades his/her gums, and the child’s head is too hot.

In an attempt to achieve a cure, all of them start with home remedies such as yoghurt, diluted tea with no added sugar, and biscuits, but some (16 of 31) avoid bananas, because they believe bananas worsen diarrhoea. Four of the 31 mothers purchase drugs (Co-trimoxazole suspension) from drug sellers if the diarrhoea does not stop. Fourteen mothers increase breastfeeding to stop diarrhoea, while 15 of them thicken bottle milk.

If diarrhoea is caused by senoon, they usually use home remedies, because they believe it is not a disease and is not caused by bacteria and that thus, there is no need for a doctor’s therapy. The 31 mothers agreed that they use what is called gutrah, which is hot oil applied to the soles, the navel, and possibly the head, for halib. A 38-year-old mother said *“This diarrhoea is not a disease; it never responds to doctors” advice, as they do not know what it is. You will jump from a doctor to the other without benefit, until you use gutrah or herbs”*. Five of the seven group members agreed by nodding their heads or saying *“yes”*. Twenty-two of the 24 mothers in the other groups believed that some types of diarrhoea are not diseases and do not respond to medical treatment. Twenty of the 31 participants expressed that many doctors do not accept or discuss these causes of illnesses.

Twenty-five of the mothers treat didan with home remedies such as seeds (e.g. Artemisia, peganum harmal) or a formula prepared by traditional healers. In addition, raqaba is treated by tarqeeb (26 of 31 mothers), which is a massage performed in a specific manner by a traditional healer or old women.

All agreed that if diarrhoea was severe or if the stool was watery or bloody, the child should be taken to a doctor. When the moderator asked about oral rehydration solution (ORS), the 31 mothers had heard about it, but only four of them said they would use it if prescribed by a doctor. The remaining 27 did not use it at all, even if prescribed by a physician. They justified their avoidance by stating that its taste is very bad and their children refuse it. When asked: *“If it is made flavoured, would you use it? ”* They replied *“Yes”*.

### Cough

The causes of cough were thought to be cold, sore throat, and allergy. All the respondents started by treating cough with home remedies such as fennel, guava leaves, egg, and ziziphus. Twelve of the 31 mothers use anti-cough syrup: herbal or salbutamol. When asked when to seek medical care, a 28-year-old mother of two children under the age of five who had a primary school education answered:

*“Cough is not serious, even if it persists for more than a week, except if it has a different sound or is productive with thick sputum that is difficult to be expelled”*.

Nineteen of the 31 mothers agreed with that belief, while a 34-year-old mother stated that she seeks medical care if the cough persists for more than three days or if the child is very young.

### Difficulty of breathing (DOB)

The mothers thought that DOB is caused by exposure to cold weather or air, allergy, inflammation in the chest, or senoon. When DOB is caused by senoon, they believed that the child would have other manifestations of senoon. A 25-year-old mother of one child under the age of five who had a primary school education said: *“I take my son immediately to the health centre even if DOB is caused by senoon, because DOB is serious and the child needs oxygen to be calm”*. Five of the eight mothers in the group agreed, while one had no answer because she had never had a child with DOB. Twelve of the 31 mothers purchase drugs for DOB (salbutamol or dexamethasone syrup).

### Attitude towards modern and traditional medicine

Thirty of the 31 mothers indicated that they start with home remedies for diarrhoea and cough. If the illness fails to resolve or if it worsens, they seek medical care. They ascribed that to what was said by a 37-year-old mother of two children under the age of five, who has a primary school education*: “As grandmothers say, traditional medicine benefits and never hurts, regardless of amount and type”*. For fever and DOB, all 31 mothers purchase drugs or consult a modern healer if the condition is severe.

When asked when they would consult traditional healers, 18 of the mothers answered that in some instances, such as if the child has ayn, the child will never improve unless treated by a religious healer (Ashaikh). Ayn is an Islamic concept believed to be caused by an evil person’s gaze (Table 2).

A 22-year-old mother added: *“If a child has strange behaviour, cries loudly, and hits his head on the walls, he should be taken to Ashaikh to read Qur’an to enforce the demon to go out of his body! This is Lafkha”*. Seven of the 31 mothers believed that lafkhais a cause of illness and participated in defining it as a local illness concept that is caused by having a demon inside a child’s body or when the child comes under the power of a demon. It occurs when he/she sits alone for a long time or goes outside during the night. A 30-year-old mother objected by stating that these behaviours are caused by extra energy in a child’s body and not by demons and that we merely take our children to Ashaikh to satisfy the grandmothers in the homes. Two of the eight said that she was right.

A 31-year-old mother of two children mentioned: *“One time my son fell down. We immediately took him to a clinic, but he did not improve, although we gave him all the prescribed drugs. Then we tried another doctor with the same result. We lastly took him to a woman to perform tarqeeb, and he started improving the same day!”*

When she was asked why she did not go back to the doctors for help, she said: *“The doctor will give us more drugs which could not cure raqaba. I was afraid that he might blame me if I told him that I’d take my son for tarqeeb. Many doctors oppose our old practices, although these are useful”*. Five of the eight women in the group discussed this statement, fully showing their support. This belief was mentioned across the four groups, and 20 of the 31 mothers supported it. A 38-year-old mother intervened: *“I think all doctors know about these methods and some of them treat their kids by tarqeeb”*.

Twenty-six respondents mentioned that they use all of the drugs prescribed by the doctors. Seven of the 31 mothers stated that they never use antibiotics for more than five days even if prescribed by a physician, because they will harm the child. By antibiotics, they mean powdered drugs that are reconstituted with water. Two mothers out of 31 said that when they go to doctors, they do not give their children all the prescribed drugs, but instead, select the most important ones, which are intravenous fluids, injections, and antibiotics.

## Discussion

The main finding from this study is that the perception of disease and the local illness concepts are crucial elements that influence treatment choices for common childhood illnesses in the city of Shuhair. There was broad agreement among the mothers in the focus groups regarding local illness concepts, and these opinions were strongly influenced the type of treatment used. Mothers know many of the medical causes of childhood illnesses, but at the same time, they attribute some illnesses to other nonmedical causes.

Similar findings were reported in studies in Yemen (Almukalla) [12], rural Ghana [16], and rural Burkina Faso [7]. The study conducted in Shuhair regarding factors affecting HSB found that considering that the illness does not need medical treatment was the second most common cause of avoiding or delaying seeking medical care.

However, that study was quantitative, and therefore, failed to explore the illness concepts behind that behaviour [6].

To explore the effects of local illness concepts on HSB, it is important to know how mothers act in response to these illnesses. The current study shows that four of the local concepts were classified as “never for medical treatment” (halib, raqaba, ayn, and lafkha), while two were considered “sometimes not for medical treatment” (senoon and didan). For each traditional concept, there was a preferred action, and while seeking a cure, it was mostly nonmedical. Medical symptoms were classified as never or sometimes “not for medical treatment” (for ‘fever and DOB’ and ‘diarrhoea and cough’, respectively). In addition, health seeking behaviour for medical symptoms was determined mainly by the type of symptoms (starting with home remedies for diarrhoea and cough, while drugs were the first choice for fever and DOB). This is similar to the findings of other studies [6,24-26]. Severity of the illness was also a stimulant for seeking medical care, and it was a frequent finding in previous studies as well [27-30].

This illnesses classification shows that these local illness concepts represent a barrier to seeking medical care. Hill et al. (Ghana) [16] found that many of the care-seeking barriers identified in his study were related to local illness concepts. Using traditional healing practices may be unfavourable if patients delay seeking medical care for treatable conditions. In addition, some practices can have harmful effects [13].

The preference of mothers to use traditional healing, whether with or without modern medicine, should motivate us, as primary care doctors and policy makers, to think of the need to add a wider range of healing options to primary care, as it seems to be desirable [31]. This is especially true for illnesses that are locally known to have an inadequate response to standard medical treatment, such as falls in children (raqaba), leading mothers to consult traditional healers, from whom they perceive they gain better results. Over the years, the World Health Organization has drawn attention to the fact that most of the populations in various developing countries around the world depend on traditional medicine for primary health care; therefore, it emphasises the fact that practitioners of traditional medicine are a potentially important resource for the delivery of health care [18].

Our focus group discussion revealed a major problem that affects HSB. The mothers tend not to disclose the local illness concepts in front of their doctors, although most of the doctors working at Shuhair Health Centre reside there and are aware of these concepts. They also do not tell their doctors when the medical treatment is not effective, but instead, jump from one doctor to another and finally treat their children traditionally. This way of communicating is not open enough, as the doctors do not take the mothers’ beliefs seriously. Physicians simply need to show non-judgmental interest and frankness when discussing traditional therapy [32]. It had been found that good patient care may necessitate the use or tolerance of both modern and traditional medicine in populations where traditional practices are popular [13]. Primary care physicians can enhance their interactions with their patients by seeking to understand their culture, particularly its approach to medicine and healing [15].

As this study coincides with the birth of the family medicine specialty in Yemen and was conducted in one of the first family medicine training centres, it is worth emphasising that family medicine is introduced to meet the health care needs of patients in the context of their communities [33]. Interventions that educate parents about appropriate health seeking and about childhood illnesses will work better if they address traditional beliefs using local terminology [16]. For example, an intervention message about halib could be: (Halib causes diarrhoea, which could lead to dehydration. If your children have halib, take them to a health facility to be assessed by the doctor for signs that prompt medical treatment).

This study has revealed mothers’ attitudes towards traditional and modern medicine. It finds that mothers prefer a combination of traditional and modern medicine, with a stronger desire for and confidence in traditional medicine. This discovery is in agreement with many other studies in Yemen and abroad [6,28,33]. There were many inappropriate behaviours regarding treatment, such as avoiding the use of ORS, using over-the-counter antibiotics as the preferred drugs for diarrhoea, and non adherence to the treatment plan, resembling the findings of previous studies [34].

Health care behaviour is the responsibility of both the doctor and the patient. The new preventive care model encourages the movement of health care providers and patients towards a non-traditional relationship in which the encouragement of healthy lifestyles by providers and the acceptance of responsibility for health behaviours by patients become the cornerstones [35].

It is important to clarify the limitations of this study. The study was restricted to the city of Shuhair; further research in other cities in Hadhramout would help confirm the generalisability of the current findings, as many beliefs are known to be common across the Hadhramout Governorate. We believe, however, that the relative uniformity of major findings across groups and the consistency of the current study’s findings with the Almukalla study [12] suggest that the findings are valid and generalisable.

As clarified in the methodology, the focus groups were not recorded, which limited the amount of data collected. In addition, including doctors in a study like this would add valuable data and answer many questions raised here. Further studies are crucial in this regard.

## Conclusions

In conclusion, local illness concepts of common childhood illnesses are widespread and agreed upon among mothers in Shuhair. They determine the type of treatment used and are associated with the avoidance or delay of medical treatment.

On the basis of the findings of this study, further research is needed in order to understand the reasons for the local illness classification system; i.e. why are some illnesses not for medical treatment? Is the reason related to the doctors (e.g. wrong diagnosis, inadequate experience), caretakers, or the illness itself? In addition, the traditional practices frequently used as a source of treatment in the community should be tested for the possibility of including them as a treatment option in primary care.

We emphasise the use of local illness concepts in intervention programs that aim to promote children’s health. Lastly, the attitudes of doctors towards traditional illness concepts should be fully explored and modified.

## Abbreviations

DOB: Difficulty of breathing; HSB: Health seeking behaviour; MDGs: The millennium development goals; MDG4: The millennium development goal 4; ORS: Oral rehydration solution; SPSS: Statistical product and service solutions.

## Competing interests

The authors declare that they have no competing interests.

## Authors’ contributions

HHW conceived the study objectives, prepared and conducted focus group discussions, and led the writing of the manuscript. ASB designed the study methodology and assisted in writing the manuscript. Both authors participated in preparing the results and approved of the final manuscript.

## Pre-publication history

The pre-publication history for this paper can be accessed here:

http://www.biomedcentral.com/1471-2458/14/581/prepub

## Supplementary Material

Additional file 1 **Evidence of RATS guideline adherence.** The file includes a point-by-point response to RATS guideline with example from our manuscript when appropriate. Click here for file
