# Supplementary material for: ‘This diarrhoea is not a disease …’ local illness concepts and their effects on mothers’ health seeking behaviour: a qualitative study, Shuhair, Yemen
Source: BMC Public Health. 2014 Jun 10;14:581. doi: 10.1186/1471-2458-14-581 (PMC4064820; doi:10.1186/1471-2458-14-581)
Supplement: Additional file 1 — Evidence of RATS guideline adherence. The file includes a point-by-point response to RATS guideline with example from our manuscript when appropriate. [file 1471-2458-14-581-S1.pdf]

'This diarrhoea is not a disease …' Local illness concepts and their effects on mothers' health seeking behaviour: A qualitative study, Shehair, Yemen

Hana H Webair[1*§], Abdulla S Bin-Gouth[1*]

[1]Department of Family Medicine, Hadhramout University, Almukalla, Hadhramout, Yemen

*These authors contributed equally to this work

[§]Corresponding author

Email addresses:

HHW: hhwebair@gmail.com

ASB: abinghouth2007@yahoo.com

R Relevance of study question

Research question explicitly stated

Research question justified and linked to the existing knowledge base (empirical research, theory, policy)

Page 5, paragraph 2:

What are the common local illness concepts in Shehair City?

How do local illness concepts affect health seeking behavior for common childhood illnesses in Shehair City?

A Appropriateness of qualitative method

Study design described and justified i.e., why was a particular method (e.g., interviews) chosen?

Page 5, paragraph 2

As the current study aimed to explore and explain people's actual thoughts and beliefs related to childhood illnesses, focus group discussions were a good method to achieve this objective because they encourage participants to talk freely and discuss topics related to their children. It also would help to develop appropriate messages for educational interventions.

T Transparency of procedures

Sampling: Criteria for selecting the study sample justified and explained

Recruitment: Details of how recruitment was conducted and by whom, Details of who chose not to participate and why

Page 5, paragraph3 & Page 6, paragraph 1&2

Visitors to the SHC vaccination unit were selected because they all have children under the age of five and are not seeking medical care for illnesses, but rather, vaccination. Another important point is that the vaccination coverage in SHC is more than 90%, which indicates that women come from almost all areas of Shehair, making the sample more representative of the overall population [6]. Mothers who are not

inhabitants of Shehair City were excluded because they may not be aware about the local concepts.

Participants

The participants were recruited purposefully between April 1 and 6, 2013, from 9:00 AM to 10:30 AM. A female community health worker approached mothers who had a child below five years of age with a history of fever, diarrhoea, cough, and/or DOB during the preceding 14 days when they entered the vaccination unit. She explained the focus group discussion and obtained verbal informed consent and contact information until the required number of participants was reached.

Data collection: Method(s) outlined and examples given (e.g., interview questions), Study group and setting clearly described,

Page 6 & 7:

Four focus group discussions were conducted, with 31 mothers participating; three groups contained eight women, and the fourth contained seven. The participants ranged in age from 20 to 38 years, with a mean age of 31 (SD±4); all of the women were married. One woman was illiterate, 19 had primary school education, and 11 had secondary school education.

Data collection and analysis

The focus groups met in a Shehair Health Centre discussion room. A female family physician acted as a moderator who facilitated, encouraged, and controlled discussions, and a female community health worker acted as an observer and took notes during the discussions. Audio- and videotaping were not used because of cultural issues and refusal of the respondents. Both the moderator and the observer had training in focus group discussions.

The discussions explored three questions for each symptom separately (Table 2): fever, diarrhoea, cough, and DOB. The questions were: "What are the causes of the illness?", "What do you do when your child has this illness?", and "What is your opinion about traditional and modern medicine?" Other additional questions were asked according to the

direction of the discussion. Probing, rephrasing, reminder questions, and hypothetical questions were used to encourage and control the discussions. A full focus group debrief was conducted after each session. The observer, the first author, and the second author were present for this meeting.

The discussions were documented and analysed using micro-interlocutor analysis, because it enables the disclosure of information regarding the level of consensus/disagreement. It helps the researcher to treat each focus group member as a unique and important study participant and provides both quantitative and qualitative information with a great deal of nonverbal language [23].

In this method, the observer took notes using paper and pencil, writing the responses in a matrix. The matrix included the questions in the rows and the participants in one column each. The responses included agreement or disagreement, manifested by both verbal and nonverbal language. Non-response was also documented.

<u>End of data collection justified and described</u>

Page 8, paragraph 3:

After completing four focus group discussions, the researchers agreed that a saturation level of ideas had been reached.

<u>Role of researchers</u>

<u>Do the researchers occupy dual roles (clinician and researcher)? Are the ethics of this discussed? Do the researcher(s) critically examine their own influence on the formulation of the research question, data collection, and interpretation?</u>

One of the researchers occupies dual roles, but the second and the independent analysts are academics and not physicians. The research methodology (including research questions) was the job of the second researcher who is not a clinician. The contribution of each author has been clarified at the end of the manuscript.

<u>Ethics</u>

Informed consent process explicitly and clearly detailed, Anonymity and confidentiality discussed, Ethics approval cited

Page 8, paragraph2:

Ethical considerations

The Department of Family Medicine of Hadhramout University reviewed and approved the study protocol. A simple and clear explanation of the research aims and procedure were provided to the SHC manager and persons involved in the study. Informed consent was obtained from the manager and staff members included in the study, and feedback was returned to them. Similarly, verbal consent was obtained from all of the mothers who participated in the discussion. The privacy and confidentiality of the respondents were ensured.

S Soundness of interpretive approach

Analysis

Indicators of quality: Description of how themes were derived from the data (inductive or deductive)
The analysis method used in this study was the micro-interlocutor analysis which has been explained above and does not include themes. The responses written in the result section were derived inductively.

Description of the basis on which quotes were chosen
Page 7, paragraph 2:

Quotes which gave answers to focus group questions and have agreed upon by several participants or that summarized statements repeated across groups were selected.

Analytic approach described in depth and justified

Analysis and presentation of negative or deviant cases

Semi-quantification when appropriate

Page7, paragraph1&2:

The observer, the first author, and the second author were present for this meeting. The discussions were documented and analysed using micro-interlocutor analysis, because it enables the disclosure of information regarding the level of consensus/disagreement. It helps the researcher to treat each focus group member as a unique and important study participant and provides both quantitative and qualitative information with a great deal of nonverbal language [23].

In this method, the observer took notes using paper and pencil, writing the responses in a matrix. The matrix included the questions in the rows and the participants in one column each. The responses included agreement or disagreement, manifested by both verbal and nsonverbal language. Non-response was also documented.

Method of reliability check described and justified
e.g., was an audit trail, triangulation, or member checking employed? Did an independent analyst review data and contest themes? How were disagreements resolved?

Page7, paragraph2:

Both authors discussed the details of the method of data analysis The first author wrote all of the results, which were then checked by the second author. Several versions were produced based on the comments from both authors. An independent doctor who has experience in focus group discussion analysis revised the results and helped in resolving disagreements.

Discussion and presentation

Findings presented with reference to existing theoretical and empirical literature, and how they contribute

This point has been followed throughout the discussion section.

Strengths and limitations explicitly described and discussed

Page17 paragraph 2 & 3:

It is important to clarify the limitations of this study. The study was restricted to the city of Shehair; further research in other cities in

Hadhramout would help confirm the generalisability of the current findings, as many beliefs are known to be common across the Hadhramout Governorate. We believe, however, that the relative uniformity of major findings across groups and the consistency of the current study's findings with the Almukalla study [12] suggest that the findings are valid and generalisable.

As clarified in the methodology, the focus groups were not recorded, which limited the amount of data collected. In addition, including doctors in a study like this would add valuable data and answer many questions raised here. Further studies are crucial in this regard.

Evidence of following guidelines (format, word count)
Detail of methods or additional quotes contained in appendix

Written for a health sciences audience

All these points have been addressed
